# Network Structure and Biological Function: Reconstruction, Modeling, and Statistical Approaches

**DOI:** 10.1155/2009/714985

**Published:** 2009-04-16

**Authors:** Joachim Selbig, Matthias Steinfath, Dirk Repsilber

**Affiliations:** 1 University of Potsdam, Department of Biochemistry and Biology, Bioinformatics Chair, 14469 Potsdam, Germany; 2 Genetics and Biometry unit, Research Institute for the Biology of Farm Animals (FBN), 18196 Dummerstorf, Germany

## 

The question of how biological function is related to the structure and dynamics of biological regulatory networks is central to systems biological research. This relationship may be observed on different scales, for example, on a global scale, or on the level of subnetworks, or motifs. Several levels exist on which to relate biological function to network structure. Given molecular biological interactions, networks may be analysed with respect to their structural and dynamical patterns, which are associated with phenotypes of interest. On the other hand, experimental profiles (e.g., time series, and disturbances) can be used to reverse engineer network structures based on a model of the underlying functional network.

Is it possible to detect the decisive network structural features with the current methods? How is our picture of the relationship between network structure and biological function affected by the choice of methods? These questions constitute the subject of the present special issue. 

The authors have approached the subject from different perspectives. Experimental data analysis focussed on specific biological problems, while simulation studies addressed more general hypotheses as well as methodological developments and comparative studies regarding the reverse engineering task. It becomes clear that these questions and the proposed answers are related and, hence, profit from an integrated presentation. 

The German Research Council (DFG) is supporting a Priority Program devoted to improve the understanding of heterosis (DFG-SPP 1149). The editors of this special issue have been working on a systems biology orientated perspective towards explaining heterosis phenomena within this framework. As part of the program, a workshop was organized in Potsdam, Germany on April, 10-11, 2008, devoted to the complex of questions described above. Ten talks were given by scientists from Sweden, Norway and Germany. The current special issue presents most contributions from the workshop and integrates them with additional contributions. 

Several authors focussed on the reverse engineering task. Hache et al. conducted a comparative study with six different reverse engineering methods based on simulated benchmark networks and profile data. Moreover, four further studies focus on improvement of special models for reverse engineering. Gao et al. propose a novel dynamic profile interaction measure. Their aim is to enable not only the evaluation of the strength, but also to infer the details of gene dependencies. Olsen et al. investigate a methodological comparison for reverse engineering by mutual information—different entropy estimators are compared in synthetic datasets and applied to real data. Song et al. reconstruct generalized logical networks to account for temporal dependencies among genes and environmental stimuli from high-throughput transcriptional data. They also compare with dynamic Bayesian network reconstruction in a simulation study and apply their approach to temporal gene expression data from the brains of alcohol-treated mice. Zeller et al. propose a reverse engineering approach with the focus on hidden variables' regulatory structure. The authors propose a Bayesian network frame for the Nested Effect Models approach to finding a hidden regulatory structure causing the observed profiles of measurable molecules.

Three contributions are concerned about how network structure in general determines the dynamics of molecular profiles they regulate. Rosenfeld in his work demonstrates through a series of simulation experiments how the pattern of pseudorandom behavior similar to "shot" noise originates from purely deterministic behavior of the underlying dynamical system. From these observation the author predicts properties of large regulatory systems in terms of the stochastic nature of their dynamics. Van Nes et al. tested the hypothesis that enrichment in certain motifs would promote dynamically stable profiles—in the case of metabolic networks. Likewise, Radde found that there is a relation between the topology of a regulatory network, and the ability of the system to exhibit certain kinds of dynamic behaviors. In her work she also proposes that modeling time delays may be an approach for improved reverse engineering.

Specific biological questions were in focus of three articles: Andorf et al. investigated a systems biological hypothesis towards explaining heterosis on the scale of metabolite partial correlation networks from Arabidopsis. They found increased partial correlations in metabolite time series profiles from heterozygote crossings and make further predictions regarding expected heterosis effects if very different lines are crossed. Ebenhoeh and Handorf describe two functional classifications of genome-scale metabolic networks—carbon utilization spectra and minimal nutrient combinations. Both strategies allow for a quantification of functional properties of metabolic networks. These are used to identify groups of organisms with similar functions. Repsilber et al. investigated how the size of a regulatory network influences its adaptive dynamics in scales of individual life-time and evolutionary adaptation. They can show that network size interacts with both types of regulatory adaptation and put specific stress on biological application examples for their model.

Schbath et al. contribute a specific theoretical work on characterizing the distribution of coloured motifs in networks. A coloured motif is a connected subgraph with fixed vertex colours but unspecified topology. Their results enable to derive a p-value for a coloured motif, without spending time on simulating null hypotheses.

From the collection of articles in this issue it has become evident, that theoretical approaches— methodological comparison studies mostly based on simulation approaches—still dominate the field. However, applications to experimental data become more and more state of the art. This fact in turn encourages also experimentally working scientists to design their experiments taking into account systems biological objectives.

Joachim Selbig

Matthias Steinfath

Dirk Repsilber

